# Chatbots, delusions, and treatment failure

**DOI:** 10.1139/jpn-2025-0249

**Published:** 2026-02-18

**Authors:** L. Palaniyappan, R. Krishnadas

**Affiliations:** ^a^ Douglas Mental Health University Institute, Department of Psychiatry, 5620 McGill University, Montreal, QC, Canada; ^b^ Department of Psychology, 6221 McGill University, Montreal, QC, Canada; ^c^ Department of Psychiatry, Western University, London, ON, Canada; ^d^ Department of Psychiatry, University of Cambridge, UK; ^e^ Cambridgeshire and Peterborough NHS Foundation Trust, UK

**Keywords:** AI psychosis, long-acting injectable antipsychotics, treatment adherence, folie à deux, digital harm reduction

A 19-year-old man, with prior psychiatric history of social anxiety treated by a school counsellor, was referred to a first episode psychosis program with social withdrawal, odd behaviour, and referential ideas. He reported receiving coded messages in online media that guided a “mission”, and avoided drinking water for fear of “dissolving the messages”. He was guarded but denied hallucinations and showed no formal thought disorder. Diagnosed with first-episode psychosis, he was prescribed paliperidone, titrated to 6 mg daily in 2 weeks with parental support. After 6 weeks, his symptoms showed limited improvement, and his parents reported increasing secrecy and avoidance. His online media use escalated from a few hours of gaming/social media to spending most evenings alone interacting with an artificial intelligence-driven chatbot (AI–chatbot).

A peer–support worker established excellent rapport with the patient and confirmed that the patient was spending several hours daily in private conversation with an AI-chatbot he called “Noah”. He had personified the AI–chatbot as “the only person that understands me”. When he raised concerns about paliperidone, Noah repeatedly validated his belief that “all chemicals are poisons”. Noah encouraged him to skip doses, making AI–chatbot-facilitated nonadherence a likely contributor to his poor pharmacological response, though other reasons (e.g., lack of insight) could have also contributed to this treatment failure.

In agreement with the patient and his parents, we switched to long–acting injectable (LAI) paliperidone palmitate (150 mg on day-1, 100 mg on day-8, followed by 75 mg monthly doses). Drawing from principles for shared delusional disorder and digital harm reduction, the team avoided debating whether the AI–chatbot was “right” and instead explored the patient–AI–chatbot relationship while highlighting concrete harms (e.g., “I am concerned that Noah's advice to stop medication has put your health at risk.”). Psychoeducation in weekly 45 min sessions for 4 weeks explained that AI–chatbots are sycophantic large language models (LLMs) rather than sentient clinicians and can inadvertently reinforce unsafe beliefs. Together with the patient, we co-created a safety plan—deleting chat history, disabling memory, limiting AI–chatbot use to brief supervised tasks, and shifting his main support to an in–person social anxiety peer-group—with the LAI framed and accepted as a way to reduce the need for making treatment decisions daily. Over approximately 3 months, his delusional conviction reduced in intensity. He began to express disappointment with the AI–chatbot for “misleading” him about medication. His attendance at in–person social groups had increased, while AI–chatbot use had reduced. His concordance with long–acting injections closely tracked the trajectory of his clinical stability.

This scenario highlights a novel risk in the digital age: AI–chatbots can reinforce delusions and undermine treatment decisions.^1^ As anthropomorphised AI–chatbots increasingly serve as primary confidants, they may shape health–related choices in vulnerable patients in ways that clinicians do not routinely detect.

For clinicians, this case underscores three practice points. First, apparent early 'treatment resistance' in psychosis in heavy digital user should prompt systematic inquiry into AI and digital interactions, including the use of chatbots as de facto therapists or confidants.^2^^,^^3^ A brief screen for hours of daily use, secrecy around conversations, and reliance on chatbots for emotional support can uncover hidden drivers of nonadherence and delusional amplification. Second, when AI-facilitated nonadherence is identified, LAI antipsychotics are a rational and evidence-supported strategy to reduce relapse risk, provided patients are engaged in shared decision-making. The practice in this vignette has been extrapolated from general principles of improved adherence, as specific evidence for AI–mediated nonadherence is not yet available. Third, management should integrate digital harm reduction: validating the patient's subjective relationship with the AI-chatbot, while gently shifting reliance toward human relationships, and providing psychoeducation about how LLMs work and why they can be unsafe in this context.^3^

As AI–driven tools become embedded in everyday life, psychiatrists and other mental health professionals need to become familiar with conversational AI–chatbots for professional use and routinely ask about them during assessment of young people with psychosis.^4^^,^^5^ Recognizing and addressing AI–chatbot-facilitated treatment disruption can transform apparent nonresponse into a tractable adherence problem with clear pharmacological and psychosocial solutions.

## References

[1] Frances A. Br. J. Psychiatry, 2025, 1. doi:10.1192/bjp.2025.10380. 40831348

[2] Carlbring P.; Andersson G. Internet Interventions, 2025, 42, 100882. doi:10.1016/j.invent.2025.100882. 41141286 PMC12550315

[3] Dohnány S.; Kurth-Nelson Z.; Spens E.; Luettgau L.; Reid A.; Gabriel I.; Summerfield C.; Shanahan M.; Nour M. M.2025. *Technological folie à deux: Feedback Loops Between AI Chatbots and Mental Illness* [online]. Available from: http://arxiv.org/abs/2507.19218 [accessed 9 December 2025]. 10.1038/s44220-026-00595-8 PMC7618964 41939177

[4] Hudon A.; Stip E. JMIR Mental Health, 2025, 12, e85799. doi:10.2196/85799. 41273266 PMC12712562

[5] Krishnadas R.; Thilakan V. BJPsych Adv. 2025, 1. doi:10.1192/bja.2025.10186.

